# An Active Learning Model for Promoting Healthy Cooking and Dietary Strategies Among South Asian Children: A Proof-of-Concept Study

**DOI:** 10.3390/nu17030541

**Published:** 2025-01-31

**Authors:** Tricia S. Tang, Simran Gill, Inderpreet Basra

**Affiliations:** 1 Division of Endocrinology, Department of Medicine, University of British Columbia, Vancouver, BC V5Z 1M9, Canada; 2 Centre for Chronic Disease Prevention and Management, Faculty of Medicine, University of British Columbia Okanagan, Kelowna, BC V1V 1V7, Canada; simran.gill@ubc.ca; 3 Barts Health NHS Trust, The Royal Hospital, Whitechapel Rd, London E1 1BB, UK

**Keywords:** South Asian, children, CVD risk factors, cooking workshop, dietary strategies

## Abstract

**Background/Objectives**: South Asian children living in Canada have a higher prevalence of cardiovascular disease risk factors compared to their non-South Asian counterparts, and poor dietary habits may contribute to this health disparity. **Methods**: This study uses a pre–post intervention design to examine the impact of a family-focused, “hands-on” cooking workshop on improving three cooking and dietary strategies: (1) using healthy cooking techniques, (2) practicing portion control, and (3) making healthy substitutions. We recruited 70 South Asian parent–child dyads (n = 140) across four elementary schools in Surrey, British Columbia. The 90 min workshop includes a didactic segment on healthy food preparation and dietary strategies, followed by an interactive cooking session where participants make a healthier version of a traditional Punjabi dish. **Results**: Among the three dietary strategies measured, both children and parents increased their frequency of using healthy cooking techniques (child *p* = 0.02; parent *p* < 0.001) and practicing portion control (child *p* < 0.001; parent *p* = 0.02). No changes were reported by either group for making healthy substitutions. **Conclusions**: Findings suggest that educational approaches that engage the family as a unit and encourage active participation are associated with improvements in cooking and dietary strategies in the South Asian community.

## 1. Introduction

In Canada, South Asians bear a greater burden of cardiovascular disease (CVD) compared to other ethnic groups [1,2,3]. Although CVD is generally diagnosed in adulthood, the atherosclerotic process itself begins early in childhood [4]. Not only is obesity a risk factor for CVD, but obesity during childhood predicts future development of CVD [5]. South Asian children living in Canada are at particularly high risk for CVD as they have a higher prevalence of overweight compared to their non-South Asian counterparts [6]. Fortunately, there is evidence that CVD risk in adulthood can be reduced by addressing elevated BMI in childhood [7]. Consequently, to reduce the burden of CVD in adulthood, prevention efforts need to start in childhood, particularly in the South Asian community.

Poor diet is a preventable risk factor for developing CVD in the South Asian community [8]. Not only is the traditional South Asian diet high in salt, fat, and carbohydrates [9,10], but this group also reports an elevated intake of full-fat dairy and ghee [11,12]. Moreover, dietary patterns worsen as a function of acculturation with increased consumption of red meat, sugar-sweetened beverages, and fast foods [10,13,14]. In addition to the quality and nutritional value of foods consumed, the methods in which meals are prepared are equally important to dietary health. For example, in South Asian culture, deep-frying, high-heat cooking, and preparing foods with ghee, partially hydrogenated fats, and reheated oils are common practices that may increase this community’s susceptibility to developing CVD [11,15]. Finally, culture can exert a strong influence on dietary patterns. The belief that non-traditional foods lack flavor or that healthy cooking techniques compromise taste may reflect culturally misinformed nutrition education [16]. Thus, interventions designed in a cultural context and involving South Asian role models are more likely to be successful [16].

Increasingly, nutrition interventions targeting youth have involved teaching children how to adopt healthier cooking methods as well as engaging learners in “hands-on” meal preparation activities. In fact, a systematic review of 42 school-based nutrition programs identified common themes of successful programs, one of which was interventions involving cooking classes. It should be noted that none of the interventions in this review were with South Asian children. However, in a systematic review and meta-analysis of 29 lifestyle interventions for South Asians, Brown [17] and colleagues found five studies examined dietary interventions for children. Of these, none included a “hands-on” component. Clearly, additional research is needed to explore interactive cooking models to improve diet-related outcomes among South Asian children, a group at high risk for developing CVD in adulthood.

For this reason, the goals of our study were to examine the impact of It’s a Family Affair, a family-focused, “hands-on” cooking workshop, to improve three cooking and dietary strategies: using healthy cooking techniques, practicing portion control, and making healthy substitutions. We hypothesized that following participation in this intervention, participants would increase their frequency in these three strategies.

## 2. Materials and Methods

### 2.1. Participants

This proof-of-concept study consisted of a 90 min cooking workshop starting with a didactic component followed by a participatory interactive cooking segment. The multi-site, single-arm, pre–post study was approved by the Behavioral Research Ethics Board (H18-02441) at the University of British Columbia and the School District in Surrey, British Columbia (BC). We recruited children in grades 3 to 6 of South Asian descent attending four elementary schools in Surrey, BC. To be eligible to participate, children had to (i) self-identify as being of South Asian descent; (ii) communicate in English, Hindi, Punjabi, or Urdu; (iii) be enrolled in one of the four target schools; (iv) be in grades 3 to 6; (v) provide parent/legal guardian consent to participate in this study. For parents, to be eligible to participate, they had to (i) have a child of South Asian descent; (ii) be able to communicate in English, Hindi, Punjabi, or Urdu; (iii) reside in the greater Vancouver area.

Recruitment strategies included providing recruitment flyers coupled with permission slips to teachers of children in grades 3 to 6. Teachers instructed students to take the flyers home for their parents to review and return a signed permission slip if interested. Research assistants then contacted interested families and enrolled them in the cooking workshop.

### 2.2. Instrumentation

On the day of the cooking workshop, parents and children completed informed consent/assent documents, administered in Punjabi, Hindi, Urdu, and English by our multilingual research staff. Assessments were conducted at baseline (pre-workshop) and at 1 month post-workshop. Surveys for parents consisted of sociodemographic information, a cooking and dietary strategies questionnaire, and a culturally tailored food screener. Children also completed all surveys but with an abbreviated demographic questionnaire.

Sociodemographic variables included the child’s age and gender, country of birth, parents’ age and gender, ethnic background, highest education level achieved, employment status, and total household income. Additionally, we also collected household information, including total household members, family history of type 2 diabetes, preferred cuisine (Western versus South Asian), time for the evening meal, and time when turning in for sleep.

Cooking and dietary strategies were assessed using a 20-item survey adapted from Raber et al.’s [18] evidence-based conceptual framework for healthy cooking. Subsequently, these authors developed a measure that demonstrated construct validity and questionnaire comprehension among a sample of 267 adults [19]. Items tapped into the following constructs: frequency (e.g., preparing meals at home, eating at fast food restaurants), technique/methods (e.g., steaming, baking, deep-frying, stir-frying), minimal usage (eating foods high in fat, sugar, carbohydrates), and additions/replacements (e.g., usage of 1% or skim milk instead of heavy cream, whole wheat flour instead of white flour). We also added items pertaining to portion control (e.g., use of measuring cups and spoons, following suggested serving sizes). Children and parents were asked how many days in the past week they engaged in these practices. Responses were categorized as never (0 days), seldom (1–2 days), sometimes (3–4 days), often (5 days), or always (6–7 days). Parents assisted their child with their responses.

Food screeners were adapted from Block and colleagues [20] and were found to be correlated with their original full-length food frequency questionnaire among 208 adults. Screeners assessed the frequency of consumption of the following food categories: fruits and vegetables (9 items), breads/wheats/grains (9 items), proteins and fats (17 items), dairy (3 items), and snacks (7 items). This survey was culturally tailored to add foods common to the traditional South Asian diet, including Indian sweets (jalebi, gulab jamun, ladoo) and snacks (samosas, pakoras, naan, roti, daals with heavy cream, mutton, etc.).

### 2.3. Procedure

The intervention was based on the social learning theory, which, in this context, posits that children learn by observing, modeling, and imitating those around them (e.g., parents, peers, instructors) and will perform the same behavior, especially if reinforced [21]. At the start of the 90 min workshop, each parent–child pair was equipped with a ‘toolkit’ that included the following items: (1) hand sanitizer, (2) measuring cups and spoons, (3) portion plate, (4) steamer, (5) non-stick frying pan, (6) low-calorie cooking spray, (7) a recipe card for “Much Better Butter Chicken”, and (8) a child-size chef’s hat. Each tool was discussed and/or utilized during the cooking workshop. The workshop was delivered once at each of the four participating elementary schools (four workshops total).

The cooking workshop began with an educational component (20 min) followed by a live, “hands-on”, interactive cooking demonstration (60 min) and ended with an orientation to healthy lifestyle resources tailored for Vancouver’s South Asian community (e.g., spaceforsouthasians.com, projectbhangra.com). The education component introduced three dietary strategies: healthy substitutions, healthy cooking techniques, and portion control. The materials for the education component were created in consultation with a dietician.

- Healthy substitutions: The traditional South Asian diet consists of foods that are high in fat, carbohydrates, sugar, and sodium. The dietician taught the parent–child pairs how to substitute for healthier ingredients. For example, 1% or skim milk instead of whole milk; canola oil instead of ghee (i.e., clarified butter); and whole wheat instead of white flour.
- Healthy cooking techniques: Traditional South Asian meals are often made using less healthy cooking techniques, including deep-frying, braising in fat, and searing in excess oil. The dietician discussed alternative methods such as steaming, baking, grilling, or broiling.
- Portion control: Exercising portion control can be difficult for many people regardless of their ethnic background. The dietician introduced the concept of ‘portion control’ and serving sizes using tools such as the portion plate, measuring cups and spoons, and the hand method for measurement.

The cooking demonstration led by a chef of South Asian descent taught parent–child pairs how to prepare a healthy version of butter chicken. During the cooking demonstration, participants applied strategies presented during the health education segment, such as substituting low-fat buttermilk for heavy cream. By observing the cooking process, the parent–child pairs learned new cooking skills and techniques, in addition to learning about the ingredients used for substitutions and their nutritional value. Moreover, the instructor provided positive reinforcement and feedback to the families, which encouraged the participants to continue practicing healthy cooking techniques. By repeatedly observing and modeling healthy substitutions, healthy cooking techniques, and portion control throughout the workshop, the families were set up to develop the habit of cooking healthy meals, which in turn will lead to better health outcomes in the long term.

### 2.4. Data Analysis

Sociodemographic characteristics were summarized using descriptive statistics for children, parents, and the total group combined. Categorical variables were reported by counts and percentages, while continuous variables were summarized as mean ± standard deviation (SD) or median and interquartile range (IQR: Q1-25th and Q3-75th). To account for the hierarchical data structure of this study [with pre- and post-workshop surveys taken on an individual participant (repeated measures nested within individuals), and child and his/her parent nested within the family], linear mixed effects models with three levels were conducted to examine the effect of the workshop on the outcome variables. In particular, individual pre- and post-workshop scores were the dependent variable; time (pre- or post-workshop) and participant status (parent or child) were independent variables. An interaction term between time and participant status was included in the model to test whether the workshop effect differed between children and parents and estimate the effect for children and parents separately. Confounding variables adjusted in the analysis were baseline score, gender, vegetarianism, and food preference. All the analyses were performed using the SAS software version 9.4. A two-sided *p*-value of 0.05 or less was defined as statistically significant.

## 3. Results

A total of 70 child–parent dyads (n = 140) were enrolled in this study. Table 1 shows the sociodemographic characteristics of participants. Sixty percent of families had more than five members living in the household; 59% reported a household income over CAD 50,000; the mean age for children was 9.0 ± 0.9, with 53% being males and 73% being born in Canada. Eighty-nine percent of parents were female, 94% were born outside of Canada, with a median of 13 years of living in Canada, and 64% had a part-time or full-time job. The majority of participants (77% children; 60% parents) identified as non-vegetarians, and 50% and 70% of children and parents, respectively, preferred South Asian cuisine.

### 3.1. Cooking and Dietary Strategies

Pre- and post-changes for three primary outcome variables are presented in Table 2. Frequency of “using healthy cooking techniques” significantly increased following the workshop for both child and parent participants (children: mean change as 0.14, 95% confidence interval (CI) [0.02, 0.25] *p* = 0.02; parents: mean change as 0.24, 95% CI [0.13, 0.35] *p* < 0.001). Similarly, significantly increased frequency for “practicing portion control” was also reported for both groups (children: mean change as 0.70, 95% CI [0.39, 1.02] *p* < 0.001; parents: mean change as 0.38 [0.07, 0.69] *p*< 0.02). No change was observed for “making healthy substitutions” for children or parents. No interaction effect of the workshop was found between children and parents for all three outcome measures. The workshop impact for the combined sample of children and parents is presented in Table 2.

### 3.2. Food Screener

Increased consumption of “green salad and non-starchy vegetables” post-workshop was observed for both child and parent participants, both having a mean change of 0.33, 95% CI [0.05, 0.61] *p* = 0.02. Reduced intake of “snacks” was reported by children with a mean change of −0.29, 95% CI [−0.42, −0.17] *p* < 0.001, but not by parents. No changes in consumption frequency of protein/fats, dairy, and South Asian breads and rice were found (see Table 2). The only between-group difference in food consumption frequency was for snack intake (interaction term: *p* = 0.01).

**Table 2:** Pre–post-workshop changes in cooking and dietary strategies and food intake.

Variable	Children			Parents			Overall	
	Pre-Workshop	Post-Workshop	Post vs. Pre [95% CI] *p*-Value	Pre-Workshop	Post-Workshop	Post vs. Pre [95% CI] *p*-Value	*p*-Value Interaction ^b^	Post vs. Pre [95% CI] *p*-Value
Cooking and dietary strategies								
Using healthy cooking techniques	2.81 ± 0.51	2.92 ± 0.43	0.14 [0.02, 0.25] 0.02	2.85 ± 0.57	3.03 ± 0.53	0.24 [0.13, 0.35] <0.001	0.20	0.19 [0.11, 0.27] <0.001
Practicing portion control	1.86 ± 1.08	2.55 ± 1.26	0.70 [0.39, 1.02] <0.001	2.16 ± 1.28	2.54 ± 1.10	0.38 [0.07, 0.69] 0.02	0.15	0.54 [0.32, 0.76] <0.001
Making healthy substitutions	3.07 ± 1.00	2.96 ± 1.00	−0.08 [−0.32, 0.16] 0.50	3.17 ± 0.98	3.20 ± 0.92	0.05 [−0.18, 0.27] 0.68	0.44	−0.01 [−0.18, 0.15] 0.86
Food intake								
Protein/fats ^1^	1.61 ± 0.25	1.59 ± 0.24	−0.01 [−0.07, 0.04] 0.59	1.54 ± 0.24	1.52 ± 0.23	−0.001 [−0.06, 0.05] 0.97	0.73	−0.01 [−0.05, 0.03] 0.68
Snacks ^2^	2.29 ± 0.71	2.01 ± 0.69	−0.29 [−0.42, −0.17] <0.001	1.53 ± 0.46	1.52 ± 0.38	−0.02 [−0.14, 0.11] 0.80	0.003	NA
Dairy ^3^	2.56 ± 0.85	2.50 ± 0.78	−0.08 [−0.27, 0.12] 0.43	1.98 ± 0.64	2.00 ± 0.78	0.01 [−0.19, 0.20] 0.95	0.54	−0.04 [−0.17, 0.10] 0.61
Green salad and non-starchy vegetable	2.33 ± 1.26	2.65 ± 1.27	0.33 [0.05, 0.61] 0.02	3.32 ± 1.09	3.64 ± 1.18	0.33 [0.05, 0.61] 0.02	0.99	0.33 [0.13, 0.53] 0.001
Fiber ^4^	1.37 ± 0.64	1.44 ± 0.62	0.06 [−0.09, 0.22] 0.42	1.80 ± 0.91	1.82 ± 0.93	0.04 [−0.12, 0.19] 0.64	0.80	0.05 [−0.06, 0.16] 0.37
South Asian breads and rice ^5^	2.72 ± 0.55	2.65 ± 0.65	−0.08 [−0.23, 0.06] 0.25	2.68 ± 0.83	2.67 ± 0.84	−0.06 [−0.21, 0.08] 0.38	0.84	−0.07 [−0.18, 0.03] 0.15

All data are presented in units of days. ^1^. Protein/fats: ground meat, beef, pork, lamb, mutton, venison, bison, duck, fried chicken, fish, sausage, cold cuts, bacon, breakfast sausage, eggs, tofu, beans, lentils, daals, daals with heavy cream, salad dressing, and spreads. ^2^. Snacks: salty and fried foods, deep-fried foods, pastries, cookies, Indian sweets, chocolate, candy, and soda. ^3^. Dairy: whole milk, yogurt, cheese, and ice cream. ^4^. Fiber: whole grain cereal and oatmeal. ^5^. South Asian breads and rice: naan, roti, paratha, poori, and rice. ^b^: interaction of time and child/parent participant.

## 4. Discussion

To our knowledge, this is the first study that found participation in a family-based cooking workshop for South Asian children and their parents. It was significantly associated with positive changes in food preparation and dietary strategies (using healthy cooking techniques and practicing portion control). Moreover, we also observed increases in the consumption of greens and non-starchy vegetables and reductions in snacking behavior. Although this was a “proof-of-concept” investigation, our favorable results could be attributed to three key elements of the intervention: being family-focused, hands-on, and school-based.

Consistent with a qualitative study examining perspectives of healthy lifestyle behaviors of 13 South Asian children and their parents [22], we also observed that family dynamics appear to play a role in food preparation and dietary patterns. Specifically, at 3 months, the frequency of adopting healthy cooking techniques and exercising portion control increased for both children and parents. However, neither of the two parties demonstrated improvements in making healthy substitutions. In other words, children model parents’ behavior and vice versa. Considering that changes (or lack thereof) were made in tandem, perhaps interventions that target the family unit rather than an individual person within the family may be more effective. In fact, a systematic review of eight randomized controlled trials (RCTs) of family-based interventions provides some evidence these models can be effective in increasing fruit and vegetable consumption, reducing intake of sugar-sweetened beverages or sugary beverages, and reducing frequency of fast-food dining [23]. For the South Asian community, family approaches can be particularly promising given the longstanding cultural values of family, shared meals, and expectations around hospitality [24].

The educational strategy of “teaching by doing” may be particularly effective for improving food preparation and dietary habits. In fact, family-based lifestyle interventions that encourage children and parents to prepare healthy meals together have been associated with improved dietary behaviors in school-aged children [25,26]. Our hands-on workshop involved child–parent teams preparing, step-by-step, a healthy version of a traditional Punjabi dish (better butter chicken) under the guidance of a South Asian chef. Each dietary strategy introduced at the start of the workshop was then applied during the active cooking component (e.g., replacing heavy cream with non-fat buttermilk and plating single serving portions). Interestingly, the use of cooking skills can translate to improved dietary behavior, as Asigbee et al. [27] found that children who are consistently involved in family cooking reported greater fruit and vegetable consumption compared to their non-cooking counterparts. Most importantly, findings from qualitative studies reveal that children express a keen interest in learning how to cook and recognize the value of developing culinary skills early on to utilize later in adulthood [28,29].

Equally important for intervention success is the setting in which programs are delivered. Several studies have demonstrated that schools are optimal locations to adopt and promote healthy dietary habits among children [30,31,32,33]. For instance, a cluster RCT of an elementary school-based program involving gardening, cooking, and nutrition education found that children in the intervention schools reported increased vegetable consumption compared to those in control schools [32]. School-based settings can be particularly important for specific populations. In fact, in a study evaluating a Bhangra dance exercise intervention with South Asian children living in the greater Surrey, BC area (the same target population as the present study), parents strongly preferred conducting sessions at the elementary schools where their children currently attended, as it offered a safe and familiar environment at no cost [34,35].

### 4.1. Implications for School Health Policy, Practice, and Equity

Schools can play a pivotal role in improving dietary health and preventing chronic disease, particularly among children from high-risk and low-resource communities. Our findings suggest that to optimize dietary interventions for South Asian children at risk for developing CVD in adulthood, not only do we need to engage caregivers/parents [36] and incorporate cultural preferences in programming [34,35], but these educational experiences need to be “hands-on” as well as conducted in the safe and low-cost setting of our schools [37]. Any lifestyle changes need to be developed, practiced, and reinforced in the environments in which the target population spends the most time. For school-aged children, these critical settings include home, school, and community-based sites. Considering that lifestyle interventions (conducted in community and school-based settings) have been demonstrated to be effective in reducing obesity-related endpoints among children of diverse ethnic backgrounds [38], this type of interactive workshop model can only add to the arsenal of public health efforts for CVD prevention.

### 4.2. Limitations

This study is not without limitations. Our cooking workshop was not developed using an evidence-based conceptual framework such as the one proposed by Raber et al. [19], which included four components: minimal usage, flavorings, ingredient substitution, and cooking techniques. Our intervention only addressed the latter two in addition to the added strategy of portion control. Second, this proof-of-concept intervention recruited a small sample size. Thus, results are not generalizable to the larger South Asian population in Canada. Moreover, this study involved a one-time workshop without ongoing sessions or a parallel home-based component. By conducting a large-scale trial as well as lengthening and intensifying the intervention, we may observe greater improvements sustained over time. Any subsequent study should incorporate post-intervention and follow-up assessments to monitor long-term maintenance of behavior change. As well, adding a qualitative component to the assessment framework will allow us to explore why and how certain healthy cooking techniques were and were not adopted. Third, because this study did not include a control condition, we cannot conclude that improvements observed were due to the intervention itself. Fourth, outcomes were measured using self-report instruments and could be subject to social desirability bias. Finally, we did not measure anthropometric or other clinical endpoints and therefore could not examine changes in CVD risk factors.

## 5. Conclusions

Childhood is the optimal time to adopt positive lifestyle habits that can lower the risk of developing CVD risk factors later in life. The goal of this study was to examine the impact of a nutrition education model that engaged both children and their parents as well as encouraged “hands-on” application of health-promoting cooking strategies. Following the workshop, children and parents increased their frequency in using healthy cooking techniques and practicing portion control. Nutrition interventions that involve the family as a unit and are conducted in the school setting may be conducive to behavior change. However, this proof-of-concept model needs to be tested in the context of a large-scale randomized controlled trial.

## Figures and Tables

**Table 1:** Sociodemographic characteristics of participants.

Variable	Total Sample (n = 140) ^a^	Child (n = 70)	Parent (n = 70)
Female sex—no. (%)	95 (67.9)	33 (47.1)	62 (88.6)
Birth country—no. (%)			
Canada	55 (39.3)	51 (72.9)	4 (5.7)
India	69 (49.3)	14 (20.0)	55 (78.6)
Other	16 (11.4)	5 (7.1)	11(15.7)
Years living in Canada (year) ^1^ Median (Q1, Q3)			12.5 (7.0, 18.0)
Languages spoken—no. (%)			
English	123 (87.9)	68 (97.1)	55 (78.6)
Punjabi	105 (75.0)	49 (70.0)	56 (80.0)
Hindi	51 (36.4)	14 (20.0)	37 (52.9)
Religion ^2^—no. (%)			
Sikh	97 (69.8)	49 (71.0)	48 (68.6)
Hindu	21 (15.1)	9 (13.0)	12 (17.1)
Other	21 (15.1)	11 (16.0)	10 (14.3)
Age (year)—mean ± SD		9.0 ± 0.9	
Grade—no. (%)			
Grade 3		34 (48.6)	
Grade 4		24 (34.3)	
Grade 5		11 (15.7)	
Grade 6		1 (1.4)	
Education ^3^—no. (%)			
Less than high school			12 (17.4)
High school graduate			18 (26.1)
Some college or 2-year college graduate			12 (17.4)
University degree			14 (20.3)
Graduate degree(s)			13 (18.8)
Employment—no. (%)			
Currently working (full-time and part-time)			45 (64.3)
Currently not working (unemployed, laid off, homemaker)			25 (35.7)
Income ^4^—no. (%)			
CAD 29,999 or less			12 (23.5)
CAD 30,000 to 49,999			9 (17.6)
CAD 50,000 to 69,999			18 (35.3)
CAD 70,000 and up			12 (23.5)
Total members in household—no. (%)			
2 to 4			28 (40)
5–6			28 (40)
7 or more			14 (20)
Personal history of Type 2 diabetes ^5^—no. (%)			1 (1.5)
Family history of Type 2 diabetes ^6^—no. (%) (yes, if anyone in the family has had Type 2 diabetes)			24 (35.8)
Vegetarian—no. (%)	44 (31.4)	16 (22.9)	28 (40.0)
Food preference—no. (%)			
South Asian	85 (60.7)	35 (50.0)	50 (71.4)
Western	28 (20)	23 (32.9)	5 (7.1)
Other	27 (19.3)	12 (17.1)	15 (21.4)
Dinnertime ^7^—no. (%)			
Less than 8 PM	87 (62.6)	45 (64.3)	42 (60.9)
8 PM or later	52 (37.4)	25 (35.7)	27 (39.1)
Bedtime—weekday ^8^—no. (%)			
Earlier than 10 PM	69 (50)	44 (62.9)	25 (36.8)
10 PM and later	69 (50)	26 (37.1)	43 (63.2)
Bedtime—weekend ^8^—no. (%)			
Earlier than 10 PM	22 (15.9)	11 (15.7)	11 (16.2)
10 PM and later	116 (84.1)	59 (84.3)	57 (83.8)

^1^: n = 4 parent participants with missing data; ^2^: n = 1 child participant ‘Refused/Prefers not to answer’; ^3^: n = 1 parent participant ‘Refused/Prefers not to answer’; ^4^: n = 19 parent participants ’Refused/Prefers not to answer’ or ’Don’t Know’; ^5^: n = 1 parent participant ’Don’t Know’; ^6^: n = 3 parent participants ’Don’t Know’; ^7^: n = 1 parent participant ‘Doesn’t eat dinner’; ^8^: n = 2 parent participant ‘Timing varies a lot depending on shifts for work’; ^a^: categories may not add up total number due to missing data.

## Data Availability

Data are available on request.
